# The Critical Contribution of Pathology Technicians to Veterinary Diagnostics, Research and Teaching

**DOI:** 10.3390/ani16162461

**Published:** 2026-08-07

**Authors:** Diana Araújo, Fátima Carvalho, Nuno Vale, Irina Amorim

**Affiliations:** 1 School of Medicine and Biomedical Sciences (ICBAS), University of Porto (UP), Rua de Jorge Viterbo Ferreira 228, 4050-313 Porto, Portugal; 2 PerMed Research Group, RISE-Health, Faculty of Medicine, University of Porto, Alameda Professor Hernani Monteiro, 4200-319 Porto, Portugal; 3 RISE-Health, Department of Community Medicine, Health Information and Decision (MEDCIDS), Faculty of Medicine, University of Porto, Rua Doutor Plácido da Costa, 4200-450 Porto, Portugal; 4 Laboratory of Personalized Medicine, Department of Community Medicine, Health Information and Decision (MEDCIDS), Faculty of Medicine, University of Porto, Rua Doutor Plácido da Costa, 4200-450 Porto, Portugal; 5 Institute of Molecular Pathology and Immunology, University of Porto (IPATIMUP), Rua Júlio Amaral de Carvalho, 45, 4200-135 Porto, Portugal; 6 Institute for Research and Innovation in Health (i3S), University of Porto, Rua Alfredo Allen, 208, 4200-135 Porto, Portugal

**Keywords:** biosafety, histotechnology, molecular diagnosis, necropsies, veterinary pathology

## Abstract

Anatomical pathology is a fundamental component of diagnostic medicine, relying on the combined expertise of physicians and technical scientific professionals to ensure accurate, reliable, and reproducible results. In veterinary medicine, the quality of pathological diagnosis directly influences clinical decision-making, treatment outcomes, and overall health management. Veterinary pathology, in particular, plays a crucial role in contemporary clinical practice. This review aims to highlight the essential role of pathology technicians within the veterinary medicine field. It explores their contributions across multiple domains, including biosafety, diagnostic workflows, quality control, research support, academic training and the challenges associated with veterinary pathology. In addition, emerging areas such as molecular pathology, digital pathology, and biobanking are discussed to illustrate the increasing scope of technical responsibilities.

## Simple Summary

Anatomical pathology plays a key role in veterinary medicine by supporting disease diagnosis and guiding clinical decisions. Within this field, pathology technicians are essential professionals involved in multiple laboratory processes, from sample handling to advanced diagnostic techniques. Despite their importance, their role is still often underrecognized, particularly in veterinary medicine. This review provides an overview of their key functions, highlights their contributions to diagnostic quality, research and education, and discusses the growing range of responsibilities in modern pathology.

## 1. Introduction

The diagnostic pathology workforce is composed of physicians and technical-scientific professionals, whose activities are essential for achieving accurate diagnoses and ensuring the quality of results [1,2,3,4]. The higher the accuracy, reliability, and consistency of these diagnostic outcomes, the more informed, timely, and effective clinical and therapeutic interventions can be implemented, thereby directly contributing to disease control, health promotion, and improved individual and population health outcomes [5].

These technical professionals emerged in the late 1960s with the development of the pathologists’ assistant role, created in response to a growing shortage of pathologists. The first training program was established in 1969 at Duke University and the Durham VA Medical Center as a one year certificate course, later extended to a two-year program [6]. In the early 1970s training initiatives were offered at the bachelor’s level; however, by the 1980s there was broad agreement that the Pathology Assistant (PathA) education should be delivered at the master’s level. Currently, some training programs are MSc-based, most of which evolved from thesis-based to course-based formats over time. As the profession became more established, accreditation and standardization gained importance, with PathAs actively contributing to this process, through the creation of professional organizations, promotion of accredited training programs, development of certification systems and examinations, and the implementation of continuing education requirements to maintain professional standards [6].

Nowadays, these professionals have a collaborative role, acting as a key interface between clinicians and pathologists by managing specimen-related issues, coordinating case triage, and supporting the implementation of specialized testing [7].

Although different designations are used across international health-care systems, the core responsibilities of these professionals are largely comparable and include the identification and implementation of biosafety measures, the performance of necropsies, gross handling and macroscopic analysis of specimens, tissue processing, and the execution of complementary techniques such as histochemistry, immunohistochemistry (IHC), and other molecular techniques, including polymerase chain reaction (PCR). Additional activities encompass slide digitization and the processing and conditioning of biological samples for biobanking purposes.

This work requires an exceptional level of technical competence and precision, underscoring the importance of specialized training combined with appropriate professional recognition. The contribution of these professionals is crucial for the production of high-quality histological sections, which form the foundation of diagnostic medical practice in anatomical pathology. Indeed, lesion diagnosis, prognostic assessment, and the definition of therapeutic options depend directly on the technical quality of histological slides. This establishes a close functional relationship between physicians and histology technicians, based on continuous collaboration in which rigorous laboratory preparation and diagnostic interpretation complement one another in a dynamic and integrated manner [1,3,4].

While the development and professionalization of pathology support personnel have been extensively documented in human medicine, similar functions are performed within veterinary pathology laboratories, where these professionals contribute to diagnostic, research, and educational activities.

Veterinary pathology constitutes an essential component of contemporary clinical veterinary practice [8]. Veterinary pathology laboratories are organized in a similar manner to human pathology laboratories, with comparable roles and responsibilities assigned to their staff [3]. This field has gained increased relevance within the framework of the One Health concept, which integrates human and animal health through the study of zoonoses and comparative medicine, while also encompassing environmental and public health [9,10]. In this context, veterinary pathology plays a central role in the diagnosis of zoonotic diseases, as well as other disorders, outbreaks, or epidemics, ranging from infections to neoplasia, which may arise as a consequence of environmental conditions shared by humans and animals [9].

Additionally, animal welfare has assumed growing importance, associated with the recognition of animals as sentient beings and the positive impact that companion animals have on human health and well-being [11]. Collectively, these factors, together with increased awareness of companion animal health and the growing tendency of owners to regard their animals as family members, have led to a significant rise in the demand for more advanced and personalized veterinary care, diagnostic investigations, and therapeutic interventions aimed at extending and improving animals’ quality of life. Consequently, there has been a substantial increase in workload within veterinary pathology laboratories [9].

This review aims to highlight the essential, yet still insufficiently recognized, role of pathology technicians in veterinary medicine, emphasizing their contribution to diagnostic quality, scientific research, and academic training, as well as the specific challenges inherent to veterinary diagnostic pathology.

## 2. Methods

This study was conducted as a narrative review of the literature. Relevant publications were identified through searches in PubMed, Scopus, and Google Scholar. The search strategy included combinations of the following keywords: “anatomical pathology technician”, “pathology technician”, “histotechnology”, “histotechnologist”, “histotechnician”, “cytotechnology”, “cytotechnologist”, “pathologists’ assistant”, “veterinary pathology”, “veterinary diagnostic pathology”, “veterinary histotechnology”, “veterinary histotechnologist”, “veterinary laboratory technician”, “veterinary laboratory professional”, “veterinary diagnostic laboratory”, “laboratory workforce”, “molecular pathology”, “digital pathology”, “biobanking”, and “translational research”.

The search focused on peer-reviewed articles, professional guidelines, educational resources, and documents from professional organizations describing the roles, competencies, training pathways, and professional frameworks of pathology-related laboratory professionals in both human and veterinary medicine. Additional references were identified through manual screening of the bibliographies of relevant publications and relevant professional organization websites.

Publications were selected based on their relevance to the objectives of the review, with particular emphasis on literature addressing the scope of practice of anatomical pathology technicians, histotechnologists, cytotechnologists, and pathologists’ assistants, as well as their contributions to diagnostic services, research, education, quality assurance, and emerging fields such as molecular pathology, digital pathology, and biobanking. Given the limited number of publications specifically addressing veterinary anatomical pathology technicians, relevant information from related laboratory professions and professional practice frameworks was also considered when applicable.

## 3. Global Organization and Professional Framework of the Pathology Workforce

There is substantial variation worldwide in the organization of pathology laboratories and in the educational and professional requirements applied to personnel working in pathology laboratories. In some regions, these professionals are formally licensed and hold university degrees in biomedical laboratory sciences, whereas in other countries less demanding qualification standards are required [12].

### 3.1. North America

In the United States of America (USA), the American Society for Clinical Pathology (ASCP) classifies this workforce into histotechnicians (HTs) and histotechnologists (HTLs), which represent the primary professional categories within the field of histotechnology. The HTs perform routine histological techniques, including fixation, processing, paraffin embedding, microtomy, and staining of human or animal tissues for subsequent evaluation by pathologists [13]. The HTLs, in turn, are qualified to perform both routine histological procedures and more complex techniques, such as immunohistochemistry and molecular methodologies, and are expected to demonstrate broader expertise in areas including management, education, and regulatory compliance within histotechnology [14].

Additionally, in the USA there is another professional designation, PathA, which corresponds to advanced practitioners trained in programs accredited by the National Accrediting Agency for Clinical Laboratory Sciences (NAACLS) and certified by the ASCP. These professionals perform duties in both surgical pathology and autopsy pathology, including macroscopic examination, post-mortem procedures, and the submission of tissues for microscopic evaluation [7,15,16,17,18]. Usually, the PathA programs last between 22 and 24 months, with the first year of the program being focused on didactic coursework, including subjects such as anatomy, physiology, embryology, general and systemic pathology, histology, medical photography, medical terminology and leadership/management; and the second year involves clinical practices, where students gain hands-on training [19].

The medical laboratory staff comprises professionals with varying levels of education and qualifications. Some roles require two-year post-secondary education, such as histotechnicians and medical laboratory technicians, while others demand a bachelor’s degree or postgraduate training, including cytotechnologists, histotechnologists, and medical laboratory scientists (MLSs) [20]. A study conducted in the USA evaluating career pathways within this workforce—including MLSs, medical laboratory technicians, histotechnologists, histotechnicians, and cytotechnologists—reported that more than half (53.9%) of MLSs hold a bachelor’s degree in Medical Laboratory Science or Medical Technology as their entry-level qualification; 4.8% possess a master’s degree and 0.3% hold a PhD [20].

In Canada, Medical Laboratory Technologists (MLTs) perform laboratory analyses and activities that contribute to disease diagnosis, treatment, monitoring, and prevention. The MLT profession encompasses histology technology, cytotechnology, anatomical pathology, and cytogenetics [21]. In Canada, to obtain PathA certification, candidates must successfully pass the certification examination of the Canadian Certification Council of Pathologists’ Assistants, organized by the PathA section of the Canadian Association of Pathologists. In addition, the University of Toronto offers a professional two-year Master of Health Science program in two clinical laboratory disciplines: Pathologists’ Assistants and Clinical Embryology [22].

### 3.2. South America

In Argentina, histotechnology is supported not only by the Sociedad Argentina de Histotecnología (SAH) [23], which promotes professional development, research, and quality standards, but also by formal technical training programs in histology that prepare professionals for specimen processing, microtomy, staining, and quality control activities within anatomical pathology laboratories [24].

In Brazil, the Sociedade Brasileira de Histotecnologia (SBH) plays a central role in professional representation, continuing education, and scientific development. The organization also supports initiatives aimed at professional regulation, recognizing specialized practice in histotechnology, cytopathology, and molecular analyses, reflecting the increasing complexity and specialization of laboratory practice [25].

### 3.3. Asia

In Japan, pathology-related laboratory practice is integrated within the regulated profession of Clinical Laboratory Technicians, which requires successful completion of a national licensing examination [26]. Medical Laboratory Science programs provide comprehensive training in disciplines such as pathology, oncology, genetics, hematology, microbiology, immunology, physiology, and clinical biochemistry. Students may pursue additional certification in cytotechnology through specialized training. These professionals are employed across a wide range of clinical, diagnostic, industrial, and research settings, with opportunities for further academic and research careers [27].

In Singapore, MLTs and laboratory scientists are trained in biomedical and laboratory sciences, preparing them to perform diagnostic analyses while ensuring quality standards. These professionals are represented by organizations such as the Association of Biomedical Laboratory Professionals [28,29].

In Hong Kong, MLTs may pursue further specialization in anatomical pathology through dedicated postgraduate training. The Chinese University of Hong Kong offers an Advanced Diploma in Pathologists’ Assistant Training, designed for professionals working in histology laboratories and focused on gross examination, specimen handling, dissection, sampling, and pathological documentation. This program reflects the growing recognition of the specialized pathology workforce [30].

### 3.4. Africa

In South Africa, pathology technologists receive profession-specific laboratory and research training. The Bachelor of Health Sciences (Honors) degree in anatomical pathology, a one-year full-time program, provides students with theoretical knowledge of the biological processes underlying disease [31]. These professionals play essential roles in diagnostic pathology services, research activities, and laboratory quality management. However, a study assessing quality management training in anatomical pathology across Africa—which included technologists from South Africa, Nigeria, and Tanzania—identified a significant need for dedicated quality management courses in anatomical pathology for pathologists, trainees, and technologists [32].

### 3.5. Oceania

In Australia, these professionals are designated as Medical Scientists and may work in anatomical pathology (histology), cytology, genetics, and molecular pathology. Professional standards and accreditation are promoted by the Australian Institute of Medical and Clinical Scientists (AIMS) [33].

According to the New Zealand Institute of Medical Laboratory Science, Medical Laboratory Technicians working in diagnostic pathology laboratories, medical research, and animal health laboratories perform sample collection, specimen handling, and laboratory testing under the supervision of scientists or pathologists. These professionals are trained through in-house, apprenticeship-style programs lasting two years and are not required to hold tertiary-level qualifications [34].

### 3.6. Europe

In Denmark, Biomedical Laboratory Scientists (BLSs) work in pathology laboratories and are responsible for the pre-analytical workflow. The bachelor’s degree in Biomedical Laboratory Science has a duration of three and a half years and provides professionals with theoretical knowledge and practical skills in biomarker analysis, validation, and quality assurance, including within clinical pathology [35]. In Finland, histotechnologists attend universities of applied sciences and obtain a bachelor’s degree in Biomedical Laboratory Science after completing 210 European Credit Transfer and Accumulation System (ECTS) credits over 3.5 years. Upon graduation, they may work in various types of clinical laboratories, including histology, hematology, clinical chemistry, and physiology [36].

In the United Kingdom (UK), the equivalent workforce mainly consists of Biomedical Scientists (BMSs), regulated by the Health and Care Professions Council (HCPC) and supported by the Institute of Biomedical Science (IBMS). Over the past decade, the IBMS and the Royal College of Pathologists (RCPath) have agreed on the formal expansion of scopes of practice in cellular pathology, including specimen dissection (Diploma and Advanced Specialist Diploma in Histological Dissection) and histopathology reporting in areas such as gynecological, gastrointestinal, and dermatopathology, using competence-based credentialing. These developments aimed to alleviate pressure and workload among pathologists, enabling reporting biomedical scientists to work within integrated teams and participate in multidisciplinary meetings [37,38].

Furthermore, BMSs performing roles similar to those of Pathologist’s Assistants have been formally authorized since 2004 to undertake specimen dissection following structured assessment and training frameworks established by professional bodies. Since then, the responsibilities attributed to PathA/BMS professionals have expanded internationally, evolving from gross examination and autopsy work to include education, laboratory management, quality assurance, research, and supervision. Overall, both pathologists and institutions increasingly recognize PathA/BMS professionals as key contributors to anatomical pathology services, education, and laboratory management [17].

In Portugal, a specialist in Anatomical Pathology, Cytology, and Thanatology (APCT) is a highly qualified health professional regulated by specific legislation within the special career of Technical Specialists in Diagnosis and Therapeutics (TSDT) [39]. This framework defines the scope of practice, educational requirements, and professional title protection. According to the Union of Senior Health Technicians (STSSs), which accurately reflects the legal definition, these professionals process biological tissues collected from living or deceased individuals; perform macroscopic and microscopic examinations using optical and electronic methods; execute and control the various stages of cytology techniques; prepare anatomical specimens for teaching and training purposes; and perform molecular techniques [40]. Additionally, they are qualified to perform clinical autopsies and to collaborate in forensic autopsies or animal necropsies. These professionals may work in public or private hospitals, anatomical pathology laboratories, research and teaching laboratories, forensic medicine units, and veterinary pathology laboratories.

About eleven public and private Portuguese higher education institutions offer accredited bachelor’s degrees in Biomedical Laboratory Sciences (BLS), enabling the acquisition of professional competencies in Clinical Pathology and Anatomical, Cytological, and Thanatological Pathology. Students and future professionals receive strong training in biosafety, macroscopic pathology, histopathology, cytopathology, molecular techniques, and laboratory quality management. However, none of these institutions currently includes mandatory curricular units specifically dedicated to veterinary pathology within their BLS programs [41,42,43,44,45,46,47,48,49,50,51]. Only two institutions offer optional curricular units with partial relevance to the veterinary context. One program includes an optional curricular unit in Animal Experimentation during the second semester of the third year, corresponding to 2 ECTS and comprising 22.5 h of theoretical-practical classes and 15 h of seminars [45]. Another institution offers an optional curricular unit in Laboratory Animal Sciences during the second semester of the second year, corresponding to 3 ECTS [41].

Despite the limited curricular integration of veterinary pathology, five of the eleven institutions refer to competencies and professional contexts applicable to veterinary medicine and explicitly mention veterinary laboratories and research centers as potential employment settings. Indeed, veterinary pathology laboratories, academic institutions, and research organizations have increasingly incorporated these professionals into their workforce, as their knowledge and multifaceted practical skills enable them to work effectively in veterinary diagnostic pathology, research, and comparative pathology. Moreover, these professionals may work abroad in European and international institutions, as access to the profession is comparable to that in other European countries and corresponds to Level 6 of the European Qualifications Framework (EQF) [52]. Considering the variability in professional designations across geographical regions, in the present review these professionals will be collectively referred to as “pathology technicians” (PTs).

## 4. Scope of Practice and Technical Areas in Anatomical Pathology

**Figure 1:**
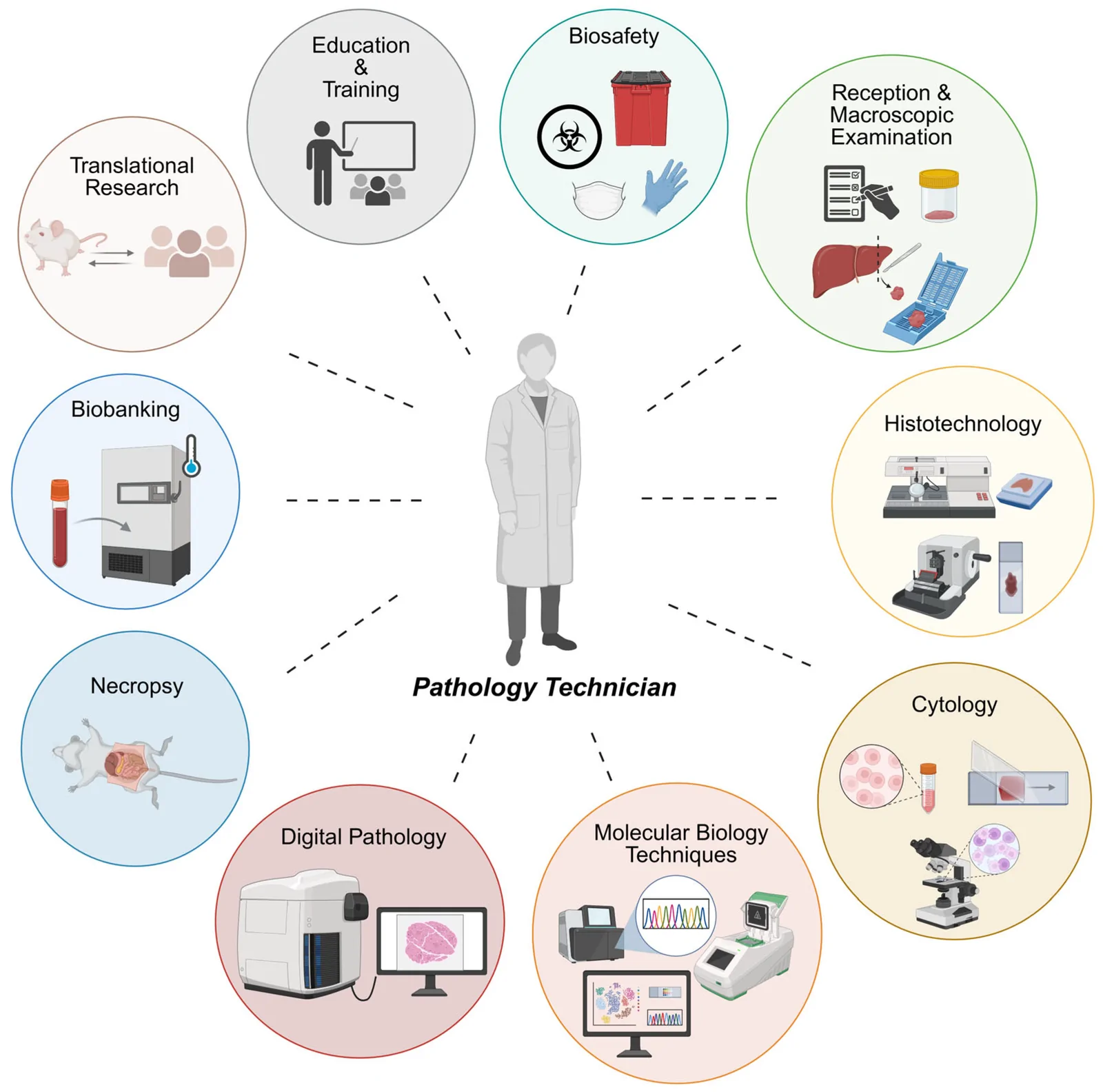
Conceptual diagram depicting the principal practice domains and core competencies of the pathology technician in veterinary diagnostics (Created in BioRender. Diana Araújo. (2026) https://app.biorender.com/illustrations/canvas-beta/6a04a5dcec35e1771cffd14f (accessed on 29 May 2026).

PTs are involved in multiple diagnostic and research activities within human and veterinary pathology. Figure 1 summarizes their core competencies in veterinary settings.

### 4.1. Biosafety

Laboratory biosafety primarily aims to prevent unintentional exposure to or release of biological agents, whereas biosecurity focuses on preventing their loss, theft, or misuse [53]. In this context, the PT plays a central role in the practical implementation of these principles across all environments in which professional activities are carried out, including the laboratory, necropsy rooms, teaching and training settings, and any other contexts involving the handling of biological material. The PT’s daily activities include the correct handling, transfer, and storage of diagnostic material, as well as the maintenance of secure workflows that prevent unauthorized access and situations of cross-contamination. In addition, these professionals are responsible for identifying and reporting instances of non-compliance with safe practices, thereby playing a decisive role in ensuring effective adherence to biosafety and biosecurity standards.

Veterinary diagnostic and research laboratories are classified by the World Health Organization (WHO) as basic laboratories, corresponding to biosafety level 2 (BSL-2), and routinely handle biological samples that may contain animal pathogens, including zoonotic agents, which pose significant occupational risks to professionals [53,54]. In this setting, the PT acts as the first line of protection within the biosafety system, relying on technical knowledge, situational awareness, and practical skills to minimize risks and ensure safe working conditions.

One of the fundamental pillars of biosafety is the correct use of personal protective equipment (PPE), the effectiveness of which depends not only on appropriate selection but also on correct donning and doffing procedures, proper storage, and safe disposal after use [53,54]. These activities are part of the PT’s daily responsibilities, as these professionals are responsible for selecting suitable PPE based on risk assessment, modeling correct PPE use for colleagues and students, ensuring the safe disposal of contaminated PPE, and identifying deficiencies or shortages that may compromise laboratory safety. As such, the PT simultaneously fulfills the roles of user, supervisor, and educator in biosafety, particularly with regard to compliance with PPE standards.

Risk assessment constitutes another essential component in determining the appropriate biosafety level and the procedures required for safe laboratory practice [53]. In routine laboratory work, it is often the PT who establishes the first point of contact with incoming specimens, performing the initial triage and identifying hazards associated with tissues, biological fluids, or carcasses. This role is critical in preventing accidental exposure, as the technician ensures appropriate handling to avoid percutaneous, mucosal, inhalation, or ingestion routes of exposure [53]. In veterinary laboratories, where human, animal, and environmental exposures must be simultaneously considered [53], the PT is directly responsible for carrying out these assessments in real time, frequently under pressure, in teaching environments, and with incomplete information, positioning these professionals at the frontline of biological hazard management.

Biological agents may be disseminated through personnel contamination, aerosol generation, waste, equipment, specimens, or animals [53]. Mitigation of these risks relies on the rigorous application of appropriate sample handling techniques to minimize aerosol formation, systematic verification of equipment functionality and decontamination, correct handling and disposal of waste according to biosafety standards, and the prevention of cross-contamination between species and cases. Through these actions, the PT safeguards the integrity and safety of all stages of the laboratory workflow.

Beyond biological hazards, veterinary laboratories also present multiple chemical and physical risks, including toxic exposures, chemical incompatibilities, and the use of sharp or cutting instruments [53]. In this domain, the responsibilities of the PT include the correct use of fixatives, solvents, and decontamination agents; the safe handling and disposal of sharps; proper labeling, storage, and management of chemicals and waste; as well as the daily monitoring of equipment and continuous assessment of procedural risks. Given that, these tasks are performed directly by PTs within pathology laboratories, their role is critical in preventing chemical and physical accidents and in maintaining a safe working environment.

Finally, effective biosafety management depends on well-defined operational controls, such as standard operating procedures (SOPs) governing pathogen handling, waste treatment, sample processing, and emergency response [54]. The PT ensures the effectiveness of these controls through consistent application in daily practice, active participation in continuous improvement processes, training of new professionals and students, documentation and reporting of incidents, and verification that emergency procedures are well understood and operationally ready for immediate implementation.

### 4.2. Reception and Registration of Biological Samples

In the context of an anatomical pathology laboratory, specimen reception constitutes a critical pre-analytical phase with a direct impact on the technical quality, safety, traceability, and diagnostic validity of examinations. It is also one of the stages in which the PT plays a central and indispensable role, as they are responsible for verifying specimen conformity at the time of arrival in the laboratory. Proper specimen identification must be immediately confirmed by the PT, who ensures consistency between the information on the container, the request form, patient data, the type of specimen, and the corresponding anatomical site [55].

Any identification errors, missing or insufficient data, or discrepancies between the request form and the specimen contents must be promptly communicated and resolved before any further processing. This step is essential to ensure traceability and diagnostic safety, thereby preventing the occurrence of irreversible errors [55].

Following this administrative validation, the PT performs an initial technical assessment, which includes verification of the type of fixative used, confirmation of an adequate fixative volume, and evaluation of container integrity to ensure the absence of leaks. Early identification of potential issues at this stage allows for the correction of pre-analytical errors or timely communication with the pathologist regarding possible diagnostic limitations [56].

Subsequently, the PT registers the specimen in the laboratory information system and assigns a unique identification number [55]. This procedure ensures full traceability throughout the laboratory workflow, an unequivocal linkage between the specimen, histological slides, and the final diagnostic report, as well as monitoring of processing turnaround times and archival control [57].

In veterinary pathology laboratories, it is essential to confirm that the submission form includes the animal’s species, breed, age, and sex. In addition, the PT must verify the total number of submitted specimens to ensure that all are properly processed and examined [8]. If any of this information is missing, or if the specimens received do not correspond to those listed on the submission form, the referring veterinary clinic must be contacted to obtain the missing data, thereby preventing subsequent errors and delays [8]. In the absence of such information, the specimen cannot be accepted by the laboratory.

This information is particularly important because different species and breeds may be predisposed to specific diseases and to varying frequencies of occurrence, making it crucial for refining differential diagnoses. With regard to sex, this parameter is also essential, as sex hormones may significantly influence the probability and risk of developing certain endocrine and neoplastic diseases [8].

Specimens submitted to a pathology laboratory typically include surgical biopsies, resection or amputation specimens, and post-mortem tissues. In all cases, the ideal specimen should be representative of the lesion and appropriately fixed [8]. Improperly collected specimens, inadequate preservation, or insufficient material compromise diagnostic evaluation and may lead to inaccurate results [58]. For this reason, the PT must verify the volume of fixative to ensure that the specimen is adequately preserved, as the fixation process preserves tissues by maintaining their architecture and preventing autolysis and putrefaction [8].

### 4.3. Macroscopic Examination

Critical decisions in histopathology are primarily made during the grossing stage, where the selection of specific areas of a specimen for processing into tissue blocks is based on tactile assessment and macroscopic features [2,59]. PTs are responsible for determining the appropriate amount of tissue per cassette to ensure optimal processing and assessing when decalcification procedures are complete [2]. The PT also ensures accurate labeling of cassettes and secondary sample containers, preventing mismatches or identification errors in subsequent processing steps.

Regarding gross examination, it is important to consider the species, the type of specimen, and the description of the samples’ anatomic location [55]. A recurrent limitation in veterinary pathology is that these details are often unspecified. These factors hinder specimen orientation and, consequently, diagnostic direction. The microscopic anatomy and disease frequencies vary by site, specifying lesion location allows the pathologist to consider particular features, and report a diagnosis more confidently in borderline cases [8]. Additionally, in cases where there is excessive hair, it should be trimmed, as it will complicate the subsequent steps of the histological processing.

Since the selection of tissue areas for processing directly determines which structures will be available for microscopic evaluation, inadequate sampling may result in loss of diagnostically relevant information. Consequently, the expertise of the PT during macroscopic examination has a direct impact on diagnostic accuracy and specimen representation.

### 4.4. Histotechnology

Most histological samples are unique and irreplaceable after acquisition and any tissue loss leads to the permanent elimination of valuable biological information [2].

In this context, fewer than 30% of all procedures are automated, and automation is not uniformly implemented across laboratories. Consequently, each stage of the histopathology workflow relies on PT judgment, with decision-making at every step directly influencing the quality of the slides. As a result, the time required to complete each step and tests may range from minutes to several hours [2]. In veterinary pathology, the technical stages of tissue processing, embedding, and microtomy are significantly challenging due to the wide range of species, anatomical structures, and tissue characteristics. These steps require continuous technical decision-making, adaptation, and precision, with the PT being essential to the quality and diagnostic value of histological preparations.

Through these stages, the PT is responsible for specimen orientation, for embedding in paraffin, deciding how deeply to section into the tissue block, and selecting which sections should be discarded or retained during the water bath stage for subsequent staining. Prior to staining, PTs are also responsible for determining whether tissue sections have sufficiently dried before heat application to prevent the formation of artifacts. They must further assess whether the histochemical procedure has reached its end point [2].

Embedding represents a critical moment in which the PT ensures that tissues are oriented in a diagnostically appropriate plane so that relevant anatomical features are exposed, since incorrect orientation may cause tissue damage and not allow an accurate diagnosis [57].

Among all sample types routinely submitted, skin is one of the most frequent tissues that presents unique challenges caused by the presence of hair. Proper orientation of skin during embedding is essential to ensure that the microtome blade encounters the tissue in the optimal plane. This relies on the PT’s technical expertise and critical judgment.

Microtomy is often the most technically demanding stage in veterinary histology because it exposes the diversity of tissue consistencies present across species. Frequent blade changes, rigorous equipment maintenance, and constant visual inspection during cutting are essential responsibilities of the PT. Through these actions, they ensure that microscopic architecture remains intact and interpretable.

The quality of tissue processing, embedding, sectioning, and staining directly influences slide quality and microscopic interpretation. Errors introduced during these stages may generate artifacts, compromise tissue morphology, or affect additional tests, highlighting the essential contribution of PTs to reliable diagnoses.

### 4.5. Cytology

PTs are able to work in cytology laboratories, and may be designated as cytotechnologists in some countries. In general, cytotechnology training demands being a medical or laboratory technologist or have completed a bachelor’s degree in biomedical sciences [60].

These professionals can perform a wide range of cytological techniques, such as sampling, processing liquid-based cytology, cell blocks, preparation and staining of samples from endoscopy and fine needle aspirates (FNA), testing for human papillomavirus (HPV), immunocytochemistry, fluorescence in situ hybridization (FISH) and other molecular techniques, attending rapid on-site evaluation (ROSE) of FNAs. In fact, a study in UK demonstrated that ROSE of FNA by BMSs improved sample quality and cellular adequacy rates for lymph node, thyroid and head and neck FNA, reducing the odds of patients to repeat the FNA or undergoing unnecessary surgery [61]. Additionally, they can prescreen cervical cytology, exfoliative cytology and FNA [60,62,63].

In veterinary diagnostic laboratories, cytology is generally integrated within the field of Clinical Pathology, where it constitutes a rapid and minimally invasive tool for the evaluation of cells obtained from tissues and body fluids. Although the sampling procedure is performed by clinicians, the technical processing of cytological material is the responsibility of the PT. Their contribution is essential to ensuring that cytological preparations maintain diagnostic quality and reliability.

Upon arrival at the laboratory, the PT is responsible for verifying proper labeling and documentation of the samples, as well as for examining them to identify potential issues, such as inadequate smear technique, insufficient cellularity, or air-drying artifacts. Veterinary cytology, similarly to human cytology, relies on rapid Romanowsky-type stains [64], with the PT being responsible for the correct execution of staining protocols and for monitoring stain quality.

Proper cytological procedures and staining are fundamental for preserving cellular morphology. Inadequate slide preparation may reduce diagnostic sensitivity and increase the risk of false or inconclusive diagnostics, making technical expertise a key determinant of cytological quality.

### 4.6. Immunocytochemistry and Immunohistochemistry

Immunocytochemistry (ICC) is performed on cytologic specimens and comprises the detection of molecules within or on cells [65], whereas IHC relies on the interaction between antibodies and their target antigens within tissue sections [65,66]. Both methods are used in the research field and as diagnostic tools, allowing the characterization of neoplastic diseases [8,65], providing prognostic evidence and enabling the detection of infectious agents [65].

One of the major challenges of immunostaining in veterinary medicine is related to the large number of species and the fact that target antigen expression is not always achieved. The same antibody can exhibit different affinities, sensitivities or cross-reactivity profiles depending on the species [67]. In fact, most of the antibodies are developed and validated for human tissues and need to be tested for activity in animal cells and tissues [68]. When performing these techniques in veterinary pathology, the PT must be aware that it can only be validated and correctly interpreted when using appropriate species-specific controls [8,65,68,69,70]. The PT must verify if the immunostaining controls have performed as expected, decisions that are critical in determining whether it should be accepted or repeated [2]. Additionally, they must be aware that IHC controls are not suitable controls for ICC. In the latter, the sample used as control should be the same as for the sample tested, which may pose a challenge due to insufficient cells [65,68].

These methods require careful optimization of multiple variables, including fixation protocol, antigen retrieval method, antibody dilution, incubation times, and quality control relies on the routine use of appropriate control slides. In veterinary immunostaining analyses, species-appropriate control tissues must be selected, comprising both positive and negative controls specifically selected to address the immunomarker under evaluation [5,65,68]. This step is of extreme importance and is sometimes underestimated. Maintaining a well-organized source of fixed tissues enables PTs and pathologists to rapidly identify appropriate control samples, thus supporting efficient and reliable immunostaining performance [5]. Accordingly, these factors require PTs in veterinary settings to develop the expertise needed to adjust ICC and IHC protocols for each species and antibody of interest [65].

### 4.7. Molecular Biology Techniques

Histotechnology has long formed the basis of pathological sciences by enabling the preservation and interpretation of tissue architecture, and it continues to play an important role in the emerging field of spatial biology. Molecular pathology aims to correlate gene expression with morphology and to use gene expression analysis to validate large sets of molecular targets [71,72]. Spatial omics technologies integrate high dimensional molecular data within intact tissue context, offering insights into biological processes and disease. Although spatial omics pipelines often emphasize advanced imaging, sequencing, and data analysis, their reliability essentially depends on tissue quality and control of pre-analytical, analytical, and post-analytical variables. These demands place PTs at the core of spatial biology, requiring exceptional precision in tissue handling, sectioning, and molecular preservation, often within limited analytical areas. Adapting traditional histological skills to complex and technological workflows highlights the need to strengthen multidisciplinary training, mentorship, and professional development, with PTs being both technical and intellectual contributors to biomedical research [73].

Next-generation sequencing (NGS) has become a cornerstone of precision oncology [74], and is especially useful in a group of well-defined but genetically heterogenous conditions, such as cardiomyopathies, connective tissue disorders, inherited retinal diseases and immunodeficiencies [75], requiring standardized workflows in molecular pathology laboratories [74]. The analytical phase, which includes all procedures from nucleic acid quantification to variant interpretation, plays a central role in ensuring the accuracy and clinical utility of molecular results. Effective NGS implementation requires a well-trained workforce and strong multidisciplinary collaboration, bringing together the expertise of pathologists and technicians, among others [74]. Despite its importance, NGS remains largely confined to specialized centers due to its dependence on specialized personnel and infrastructure. However, advances in automation enable the integration of NGS within diagnostic histopathology laboratories, where PTs increasingly contribute to sample preparation, workflow optimization, and quality control, facilitating the integration of molecular and morphological data and improving diagnostic efficiency [76]. Once again, this new reality highlights the growing importance of PT expertise in connecting traditional histopathology and advanced molecular diagnostics within precision medicine.

PTs are also a key contributor in supporting quality improvement, quality assurance, and quality control processes within pathology laboratories. These responsibilities include maintaining optimal pre-analytical conditions, such as minimizing cold ischemia time, which is critical for preserving tissue integrity and ensuring reliable diagnostic outcomes [18,77].

Biomarkers, defined as measurable indicators of biological processes, disease states, and responses to therapeutic or environmental interventions, may be molecular, histological, radiographic, or physiological in nature. This definition highlights the importance of histotechnology and anatomical pathology in supporting reliable diagnostic and research applications across species [73]. PTs contribute significantly to biomarker research and precision diagnostics; however, their role remains underrecognized due to traditional perception of their work as purely technical. Increasing their visibility through academic contributions and interdisciplinary collaboration is essential to launch them as key partners in modern research, where these professionals’ expertise and knowledge can play a critical role in advancing precision medicine [78].

Regarding veterinary medicine, the increasing use of biomarkers in animal health underscores the relevance of tissue-based analyses in this field [73]. Molecular biology techniques have become increasingly relevant in veterinary pathology [79,80], although their routine diagnostic use remains limited due to high cost, regulatory constraints, specialized equipment, the need for standardized bioinformatic pipelines and the establishment of validation frameworks, and the requirement of laboratory quality standards [79,80,81]. These methods are primarily applied in research settings, outbreak investigations, and translational or comparative medicine projects [79]. Nevertheless, NGS has demonstrated value in veterinary medicine for pathogen identification, characterization of genetic diseases, assessment of microbial variety and monitoring of infectious disease spread [80].

Within this context, the PT plays an essential role, contributing through technical expertise that supports molecular investigations conducted in diagnostic and research laboratories.

### 4.8. Digital Pathology

In recent years, there has been an increase in digital pathology (DP) applications worldwide, requiring hardware and software, such as scanners, whole slide imaging (WSI) and artificial intelligence (AI) [35,82]. This technology can be used for histologic diagnosis, in the research field, or in the academic environment [82]. The implementation of DP must comprise a multidisciplinary team of pathologists, PTs and management professionals [35,82].

A Danish study reported that PTs will play a key role in the transition of conventional to digitalized diagnostic workflow [35]. Therefore, it is of major importance to adapt undergraduate and postgraduate educational courses to support and provide the PT with the necessary competences [35].

DP has progressed in veterinary medicine. The WSI, telepathology, and AI-supported image analysis are increasingly integrated into diagnostic workflows, research, and teaching. Several veterinary laboratories and academic institutions have already incorporated virtual microscopy as an essential tool, with many transitioning from traditional optical microscopy to fully digital slide viewing [83].

As veterinary pathology laboratories adopt digital workflows, the PT emerges as the main worker ensuring the reliability, accuracy, and efficiency of digital systems. This work encompasses operating WSI scanners; selecting appropriate scanning parameters; and monitoring scan quality. It is known that the precision of telepathology and AI-assisted diagnostics relies on high-quality scanned images, which demonstrates the technician’s contribution in this setting [83].

As DP depends on high-quality slide preparation and scanning, PTs play a crucial role in ensuring image quality and reproducibility. Technical deficiencies acquired before or during digitization may compromise subsequent image analysis and diagnostic interpretation.

### 4.9. Necropsy Procedures

Necropsies are a critical component of veterinary diagnostic pathology, determining the cause of death, supporting disease surveillance, forensic investigations, and teaching activities [84]. Although necropsies are performed under the authority of a veterinary pathologist, the PT plays an indispensable operational and technical role, since they ensure both the scientific quality and biosafety procedures. Necropsies involve diverse animal species, anatomical complexities, and zoonotic risks, which claim a high level of technical skills and coordination. The PT is responsible for organizing all necessary equipment, instruments, PPE, disinfectants, and sampling containers.

In veterinary pathology, necropsied animals vary significantly in size, requiring different approaches and technical skills. In field necropsies of large animals, the selection of an appropriate necropsy site must prioritize practicality, operator safety, and biosecurity, and should be carried out in locations that allow effective disinfection or restrict access by other animals [84]. In this context, the PT often assists the veterinary pathologist by performing the initial opening of the carcass, stabilizing the animal, exposing major organs, and facilitating efficient dissection.

Throughout the necropsy, the PT plays a dynamic support role, such as managing instruments and reagents and preventing cross-contamination between body regions. Moreover, veterinary necropsies frequently require sample collection not only for histopathology but also for microbiology, virology, parasitology, toxicology, and molecular diagnostics, which must be collected in appropriate containers, correctly labeled, and preserved according to the protocols suitable for each diagnostic method.

At the end of field necropsies, the PT is frequently in charge of closing the carcass, disinfecting tools and work surfaces and preventing environmental contamination. These actions are important to control disease spread and maintain environmental safety, aligning with veterinary biosafety principles.

The PT’s responsibilities extend beyond the necropsy room or field, as they may also coordinate with histology, microbiology, toxicology, and other laboratory units. In forensic necropsies, their role is particularly critical, as strict maintenance of the chain of custody must be ensured throughout the entire procedure. In academic settings, the PT plays a relevant pedagogical role, by modeling correct biosafety behavior for veterinary students; supervises PPE compliance during necropsy classes; assists students in learning necropsy techniques, including organ examination and identification; teaches proper sample collection for each diagnostic technique; helps students in preparing cytology smears from cadavers and performing rapid stains. Through these actions, the PT contributes to the training of future veterinarians, promoting the adoption of biosafety and diagnostic sampling skills, namely day one competencies, among veterinary students.

### 4.10. Biobanking Support and Sample Management

Biobanks emerged to address the demand for high-quality biological samples in biomedical research and have evolved to support a wide range of developing fields, such as proteomics, genomics, and personalized medicine [85].

In this scenario, the most challenging tasks involve the guarantee of large-scale collection of samples, but also ensuring their preservation through the development of standardized procedures that maintain the integrity of different types of samples [86]. Therefore, the staff working at a biobank must be trained and experienced on histopathology techniques and specimen processing in order to efficiently manage the numerous and different types of samples that will be used for both clinical and research aims [87,88].

Institutions with large-scale tissue banking operations increasingly recognize the value of dedicated PTs in biobank management. In addition to supervise tissue collection, they contribute to specimen tracking and quality data management, while also supporting personnel recruitment and budget planning [18].

Veterinary biobanks have gained interest due to their contributions to veterinary research and translational medicine, since it collects and stores samples for future scientific use by academic researchers and industry entities [89].

In this context, the PT represents an essential operational role, being the basis of the biobanking workflow, as their expertise ensures that samples are properly collected, processed, preserved, cataloged, and distributed under strict quality and traceability standards. High-quality biobank specimens depend on rigorous pre-analytical handling.

## 5. Beyond Routine Pathology Workflow

Besides the functions of the PT in the pathology laboratory, these professionals demonstrate competence in other advanced laboratory techniques, reflecting the multifaceted and versatile nature of these professionals, including, for example, electron microscopy, cell culture techniques and flow cytometry.

Due to their specialized training, these professionals are well qualified to take on supervisory and management roles within several laboratories. In fact, most of them already perform such functions, including personnel management, workflow optimization, protocol revision, staff scheduling and training, and supervision of daily laboratory activities. Their responsibilities may also be extended to equipment maintenance, quality control, inventory management, specimen tracking, and the evaluation of staff performance and competencies. Moreover, PTs with strong leadership and organizational skills may even be considered for higher administrative roles, where responsibilities include financial planning, quality assurance, safety supervision, human resource management, strategic development of laboratory services, and compliance with accreditation requirements [7,18].

Beyond their main responsibilities and tasks previously mentioned, PTs contribute substantially to the educational task, research production, and innovation of veterinary pathology laboratories. Their professional activities extend into multiple domains that support both academic institutions and the broader veterinary research community, reinforcing their role as indispensable members of the diagnostic and scientific workforce.

### 5.1. Education and Training

Many histotechnology programs face shortages of qualified faculty and staff, with educators often being recruited for their technical expertise rather than formal training in teaching. As a result, not all mentors are equally prepared to deliver content successfully or assume leadership roles. These challenges are amplified by heavy workloads, as educators frequently combine teaching responsibilities with routine laboratory duties, and by the limited availability of dedicated educational resources, including textbooks and standardized learning materials in histotechnology [90].

Despite these difficulties, teaching histotechnology offers important professional rewards, since mentors are expected to maintain up-to-date knowledge, reinforcing their own technical skills, and many actively seek to address gaps in their own expertise. On the other hand, it allows close interaction with students, promoting strong mentorship and engagement in their academic and professional development [90].

Veterinary pathology departments frequently rely on PTs to support the practical training of undergraduate and postgraduate students. This includes students enrolled in veterinary medicine degrees, and other life-science disciplines, providing direct mentorship in grossing, histologic processing, microtomy, histochemistry and IHC.

In fact, the teaching of biomedical sciences in veterinary medicine has advanced to integrate knowledge with clinical application, which is essential for the development of clinical, diagnostic and therapeutic decision-making, and evidence-based practice. These competencies are supported by a range of practical skills, such as necropsy, anatomical dissection, cytology, microbiological, virological, and parasitological testing, which are traditionally acquired through laboratory-based training [91].

In this educational role, the PTs act as technical and pedagogical specialists, supporting the development of future PTs, veterinarians, and researchers.

### 5.2. Translational Research

In translational research, histology laboratories play a central role by supporting experimental workflows, providing technical expertise, and advising research investigators. Their contributions range from routine tissue processing to troubleshooting IHC assays, placing histology at the interface between molecular and biochemical research and the study of disease processes. PTs contribute specialized skills to meet diagnostic demands and, in research environments, serve as key collaborators who must adapt to the various and evolving challenges inherent to research projects [5].

Animal models are frequently used in research and histology laboratories play a crucial role in this process by enabling the assessment of pathological alterations and phenotypic characteristics [5].

As animal models are a major part of biomedical and translational research, veterinary pathology laboratories are essential for the precise assessment of tissue alterations and animal disease, highlighting the critical contribution of PTs with experience in veterinary pathology to constitute robust and clinically meaningful research data, since the reproducibility and reliability of translational research depend on standardized tissue handling, processing, and quality control procedures.

## 6. Future Perspectives

As veterinary pathology continues to evolve, the role of PTs is expected to become increasingly specialized and multidisciplinary. Although many competencies are shared with human pathology, veterinary pathology presents unique challenges related to species diversity, anatomical variation, necropsy procedures, the handling of a wide range of biological specimens, and the integration of comparative pathology. Consequently, veterinary pathology requires expertise that extends beyond the competencies traditionally acquired through general anatomical pathology training.

Greater consideration should be given to the inclusion of veterinary pathology-related content within the curricula of undergraduate pathology technician training programs. While current educational frameworks provide a strong foundation in anatomical pathology, greater exposure to veterinary-specific topics—including comparative pathology, species-specific anatomy and pathology, necropsy techniques, biosafety procedures, quality assurance, and the handling of animal-derived specimens—would better prepare graduates for the distinctive challenges of veterinary pathology and facilitate their integration into veterinary diagnostic, research, and academic settings. Such curricular developments would also help promote veterinary pathology as a relevant and attractive professional pathway for future PTs.

Building on this foundation, future professional development and postgraduate training opportunities should place greater emphasis on advanced veterinary pathology competencies, ensuring that PTs are adequately prepared to work across diagnostic, research, and academic environments. The establishment of specialized training pathways and continuing education programs would contribute to the development of a highly qualified and adaptable workforce capable of responding to the evolving demands of the profession.

Furthermore, the increasing implementation of molecular diagnostics, digital pathology, artificial intelligence, spatial omics, and biobanking is expected to further expand the scope of practice of PTs. These advances will require ongoing professional development and the acquisition of new technical and analytical competencies. To ensure that PTs remain key contributors to diagnostic excellence, translational research, and veterinary education, it will be essential to promote opportunities for specialization, lifelong learning, and interdisciplinary collaboration.

Future developments should also encourage greater international harmonization of competencies, training standards, and professional roles for PTs working in veterinary pathology. Such harmonization would strengthen professional recognition, facilitate mobility and collaboration across institutions and countries, and support the adoption of best practices within veterinary pathology laboratories.

Finally, the growing importance of the One Health concept highlights the valuable contribution of PTs at the intersection of animal health, human health, and biomedical research. Their involvement in disease surveillance, comparative pathology studies, translational medicine, biobanking initiatives, and multidisciplinary research projects is likely to become increasingly important. As veterinary pathology continues to advance, PTs will play a crucial role in supporting innovation, diagnostic quality, and scientific progress, reinforcing their position as indispensable members of modern veterinary pathology teams.

## 7. Conclusions

Pathology technicians play a fundamental and multidimensional role in veterinary pathology. Across all stages of the diagnostic process, from specimen reception to the final reporting of results, their actions directly influence the quality, accuracy, and reliability of diagnostic outcomes. These professionals ensure procedural rigor and biosafety compliance, adapt laboratory protocols to species-specific requirements, and contribute decisively to the production of histological, cytological, and immunohistochemical preparations of high diagnostic value.

Beyond routine diagnostic activities, PTs also make substantial contributions to veterinary research and education. Through their involvement in laboratory training, supervision of practical activities, implementation of quality assurance procedures, and promotion of biosafety standards, they support the development of essential competencies in veterinary students and early-career researchers while contributing to the generation of reliable scientific data and high-quality research outputs.

Despite their technical, scientific, and educational relevance, the visibility and recognition of PTs within veterinary pathology remain comparatively limited. Their contributions are frequently perceived as primarily technical, overlooking the specialized expertise, professional judgment, adaptability, and responsibility required to support complex diagnostic, research, and teaching activities.

This review highlights the breadth and significance of the PT’s role within contemporary veterinary pathology and underscores their value as integral members of multidisciplinary teams. Their contributions extend far beyond laboratory processing, encompassing diagnostic support, research advancement, educational activities, and the maintenance of quality and biosafety standards. As veterinary pathology continues to evolve, recognizing and valuing the expertise of PTs will be essential to sustaining excellence in diagnostics, fostering innovation, and strengthening the scientific and educational missions of veterinary pathology institutions.

## Data Availability

No new data were created or analyzed in this study. Data sharing is not applicable to this article.

## Abbreviations

The following abbreviations are used in this manuscript:

IHC	Immunohistochemistry
PCR	Polymerase Chain Reaction
USA	United States of America
ASCP	American Society for Clinical Pathology
HTs	Histotechnicians
HTLs	Histotechnologists
PathA	Pathologists’ Assistant
NAACLS	National Accrediting Agency for Clinical Laboratory Sciences
MLS	Medical Laboratory Scientists
MLTs	Medical Laboratory Technologists
BLSs	Biomedical Laboratory Scientists
ECTS	European Credit Transfer and Accumulation System
UK	United Kingdom
BMSs	Biomedical Scientists
HCPC	Health and Care Professions Council
IBMS	Institute of Biomedical Science
RCPath	Royal College of Pathologists
APCT	Anatomical Pathology, Cytology, and Thanatology
TSDT	Technical Specialists in Diagnosis and Therapeutics
STSSs	Union of Senior Health Technicians
EQF	European Qualifications Framework
PTs	Pathology Technicians
WHO	World Health Organization
BSL-2	Biosafety Level 2
PPE	Personal Protective Equipment
SOPs	Standard Operating Procedures
FNA	Fine Needle Aspirates
HPV	Human Papillomavirus
